# Transcriptome Profiling of the Potato (*Solanum tuberosum* L.) Plant under Drought Stress and Water-Stimulus Conditions

**DOI:** 10.1371/journal.pone.0128041

**Published:** 2015-05-26

**Authors:** Lei Gong, Hongxia Zhang, Xiaoyan Gan, Li Zhang, Yuchao Chen, Fengjie Nie, Lei Shi, Miao Li, Zhiqian Guo, Guohui Zhang, Yuxia Song

**Affiliations:** 1 Ningxia Key Laboratory for Agrobiotechnology, Agricultural Bio-Technology Center, Ningxia Academy of Agriculture and Forestry Science, Yinchuan, Ningxia Hui Nationality Autonomous Region, China; 2 National Key Laboratory of Plant Molecular Genetics, Shanghai Institute of Plant Physiology and Ecology, Chinese Academy of Sciences, Shanghai, China; 3 Guyuan sub-centers of National Potato Improvement Center, Ningxia Academy of Agriculture and Forestry Science, Guyuan, Ningxia Hui Nationality Autonomous Region, China; Institute of Crop Sciences, CHINA

## Abstract

Drought stress can seriously affect tuberization, yield and quality of potato plant. However, the precise molecular mechanisms governing potato stolon’s response to drought stress and water supply are not very well understood. In this work, a potato (*Solanum tuberosum* L.) variant, Ningshu 4, was subjected to severe drought stress treatment (DT) and re-watering treatment (RWT) at tuber bulking stage. Strand-specific cDNA libraries of stolon materials were constructed for paired-end transcriptome sequencing analyses and differentially expressed gene (DEG) examination. In comparison to untreated-control (CT) plants, 3189 and 1797 DEGs were identified in DT and RWT plants and 4154 solely expressed DEGs were screened out from these two comparison groups. Interestingly, 263 genes showed opposite expression patterns in DT and RWT plants. Among them, genes homologous to Protein Phosphatase 2C (PP2C), Aspartic protease in guard cell 1 (ASPG1), auxin-responsive protein, Arabidopsis pseudo response regualtor 2 (APRR2), GA stimulated transcripts in Arabidopsis 6 (GASA6), Calmodulin-like protein 19 (CML19), abscisic acid 8'-hydroxylases and calcium-transporting ATPase, et al. were related with drought-stress and water stimulus response. Sixteen DEGs involved in starch synthesis, accumulation and tuber formation exhibited significantly different expression upon re-watering. In addition, 1630, 1527 and 1596 transcription factor encoding genes were detected in CT, DT and RWT. DEGs of ERF, bHLH, MYB, NAC, WRKY, C2H2, bZIP and HD-ZIP families accounted for 50% in three comparison groups, respectively. Furthermore, characteristics of 565 gene ontology (GO) and 108 Kyoto Encyclopedia of Genes and Genomes pathways (KEGG) were analyzed with the 4154 DEGs. All these results suggest that the drought- and water-stimulus response could be implemented by the regulated expression of metabolic pathway DEGs, and these genes were involved in the endogenous hormone biosynthesis and signal transduction pathways. Our data provide more direct information for future study on the interaction between key genes involved in various metabolic pathways under drought stress in potato.

## Introduction

Global climate change is reducing the reliability of rainfall, the availability of soil-water, and consequently, limiting plant production. Potato (*Solanum tuberosum* L.) ranks as the fourth most predominant non-grain food crop in the world. Due to their shallow root system, which limits water extraction from soil, potato plants are sensitive to drought stress. Leaf size, photosynthesis rate, tuber number, yield and quality were all severely limited when grown under a drought stress condition [1, 2]. Therefore, in order to breed new potato varieties with improved drought resistance, the molecular mechanisms governing water use, tuber starch synthesis and accumulation under drought conditions must be defined.

In higher plants, drought stress can induce a wide range of physiological and biochemical responses regulated by genes encoding functional and/or regulatory proteins, to maintain a normal homeostasis and avoid wilting death [3]. Fast and efficient recovery from water stress will allow agricultural crops to adapt to the changes of meteorological conditions and maximize their growth and production during drought stress. In a diploid potato population, 47 quantitative trait loci were identified, out of them, 28 were drought-specific, and 17 were specifically expressed during recovery from drought stress [4]. Recently, the published potato genome [5] and transcriptome [6] with corresponding gene structure, location, and functional annotation provide powerful resources for the understanding of the complex responses to drought stress in potato. However, data currently available on transcriptomic changes upon rehydration in potato is insufficient, and the precise pathways involved in drought responses and recovery from water deficit still remain to be deduced.

Transcriptome sequencing technologies provide a framework dataset for researches related to construction of transcriptome map, determination of metabolic pathways, clarification of gene expression patterns, and mining of new genes. To explore the molecular regulation mechanisms of plants in response to different stresses, transcriptome analysis has been performed on a variety of crops, including sunflower (*Helianthus annuus*) [7], grape (*Vitis vinifera*) [8], maize (*Zea mays*) [9] and sorghum (*Sorghum bicolor* L. Moench) [10]. Data reported in these studies have provided information on the networks of regulatory and functional genes at different levels.

Upon the completion of whole genome and transcriptome sequencing of potato DM1-3 516R44 [5, 6], a number of genes related to drought-stress response and water use efficiency have been identified by quantitative trait locus mapping [2, 4], and transcriptome analyses of stress response in potato leaves has been performed with plants grown under a single condition. However, molecular mechanism of potato plant, especially the tuber or stolon responsing to water-stimulus are not very well understood with the results available now. Tubers are the main “sink” organ and it also the most important agronomic trait of potato plants. In addition, the tuber bulking stage is a critical period of time for potato tuberization, during which plants are extremely sensitive to water deficit. Therefore, we explored the molecular mechanisms underpinning drought stress responses in the stolon tissue of a potato variety. Plant cultivated under severe drought stress condition during tuber bulking stage were used for next-generation transcriptome sequencing to fully resolve gene expression profiles in response to water deficit and re-watering.

## Materials and Methods

### Plant materials and drought treatments

The potato (*Solanum tuberosum* L.) strain Ningshu 4 was from our own lab. All plant materials were collected from our Guyuan Experimental Field (Guyuan, Ningxia Hui Autonomous Region, China), which does not involve any endangered or protected species. Before tuber bulking stage, 40 healthy and uniform ball-plants were transplanted into buckets and cultivated indoors at room temperature under 12-hr light/dark cycles at 4,000 lux. Experimental soil was collected from a test site in Guyuan City, Ningxia Hui Autonomous Region, with the maximum field water-holding capacity of 21%.

At flowering stage, the above plants were split in to three groups with ten plants in each group. The first group of plants was exposed to severe drought stress treatment (DT) for three days, during which relative soil moisture was controlled at 35–40% of the maximum field water-holding capacity. The second group of plants was exposed to severe drought stress treatment for 3 days, followed by re-watering to the control level and grown for 3 days (RWT). And the last group of plants was maintained under normal watering condition throughout the time period as control (CT). Then, stolon tips from three randomly selected plants of each group were pooled together, immediately grounded into fine powder in liquid nitrogen for sequencing analysis.

### Total RNA extraction

Total RNA was extracted from plant materials using a TransZol Plant kit (ET121-01, Transgen Biotech, Beijing, China) according to the manufacturer’s instruction. Digestion of gDNA in each sample was conducted using DNase I (2212, TaKaRa, Dalian, China). The integrity and purity of RNA was confirmed using 1.2% non-denaturing agarose gel electrophoresis and the Agilent 2100 RNA 6000 Kit (Agilent Technologies, Santa Clara, CA, USA).

### Strand-specific cDNA library construction and sequencing

From each sample, 3 μg of total RNA was taken to construct strand-specific cDNA libraries using the NEBNext Ultra RNA Library Prep Kit (New England labs, Ipswich, MS, USA). The quality of cDNA libraries was tested, and quantified cDNA was subjected to purification, elution, end repair, poly(A) addition and adaptor ligation. Then, agarose gel electrophoresis was used for size selection of gene fragments, followed by PCR amplification. The established cDNA libraries were sequenced using the Illumina Hiseq 2000 platform (Illumina Inc., San Diego, CA, USA) to generate 100-bp paired-end reads.

### Sequence analysis

Image data obtained from cDNA sequencing was converted to the corresponding nucleotide sequence data. Raw reads were firstly processed through in-house perl scripts. In this step, clean data (clean reads) were obtained by removing reads containing adapter, reads containing ploy-N and low quality reads from raw data. At the same time, Q20, Q30, GC content and sequence duplication level of the clean data were calculated. All the downstream analyses were based on the clean data with high quality. Then, index of the reference genome (PGSC_DM_v3_2.1.10) was built using Bowtie v2.1.0 and paired-end clean reads were aligned to the reference genome using TopHat v2.0.9 (Broad Institute, Boston, MA) [11]. Next, HTSeq v0.5.3 was used to count the reads numbers mapped to each gene. And RPKM [12] of each gene was calculated based on the length of the gene and reads count mapped to this gene. All the clean data have been deposited in the Short Read Archive (SRA) at the NCBI database with the project accession number SRP056128.

### Screening, clustering and functional annotation of differentially expressed genes (DEGs)

DEGs between different treatments were identified based on the RPKM value calibrated with the edgeR program [13]. DEG analysis was conducted using the DEGSeq R package, as described previously. A q value <0.005 and a fold change |log2| ≥1 were used as the screening cutoffs for extremely significant differential gene expression between two samples.

Following pairwise comparison of samples, gene ontology (GO) classification of DEGs was performed using the GO seq software [14] with corrected p-Value <0.05 as the screening cutoff for enriched GO terms. GO enrichment was analyzed in the categories of molecular function, cellular component and biological process. Enrichment analysis of Kyoto Encyclopedia of Genes and Genomes (KEGG) metabolic pathways was performed using KOBAS [15].

### Quantitative real-time PCR (qRT-PCR) verification of DEGs

To test the validity and accuracy of transcriptome sequencing data, 10 genes were randomly selected from sequencing data for fluorescent qRT-PCR. Primers were designed using Primer Express software (v3.0, Applied Biosystems), and primer sequences were shown in S1 Table. The *elongation factor 1-α* (*ef1α*) was used as a reference gene [16]. The 20 μl PCR reaction buffer contains: 2 μl diluted cDNA, 1 μl each of forward and reverse primers (10 μmol·L^-1^), 10 μl qPCR Master Mix (ABI, Foster City, CA, USA) and 6 μl ddH_2_O. Amplification of genes was performed on a LightCycler 480 Real-Time PCR machine (Roche, Basel, Switzerland) under the following conditions: 95°C for 3 min, followed by 40 cycles at 95°C for 15 sec and 60°C for 40 sec. Detection of each gene was repeated 3 times. The 2^-(ΔΔCt)^ method [17] was used to calculate and calibrate the expression level of target genes in different treatments.

## Results

### Total RNA quality testing

The quality of total RNA extracted from the collected stolons of CT, DT and RWT plants was tested using agarose gel electrophoresis and an Agilent 2100 Bioanalyzer. All the three samples had 28S:18S ratios in a range of 1.8–2.0 with intact 28S, 18S and 5S RNA bands, high RNA purity, and a mean RNA integrity number (RIN) >8.0, which met the requirements for library construction and sequencing.

### Sequencing data and DEG analysis

After transcriptome sequencing, a total of 78448498, 74764759, and 71081020 raw reads were obtained from CT, DT, and RWT cDNA libraries, respectively. From these reads, 21.74 G clean bases were obtained by filtering impurities, and 76.1% of them fell into the published potato genome. Sequencing analysis of CT, DT and RWT cDNA libraries produced 46302, 47166 and 46341 genes, and 75924, 76516 and 74678 transcripts, respectively (Table 1).

**Table 1:** Statistical analyses of cDNA libraries from potato stolons under different growth conditions.

	CT (Control)	DT (Drought stress treatment)	RWT (Re-watering after drought stress treatment)
Raw reads	78448498	74764759	71081020
Clean reads	74826108	71748174	68670240
Total mapped	56185839 (75.09%)	52476932 (76.42%)	56769533 (76.8%)
Clean bases (G)	7.48	6.86	7.4
Error rate (%)	0.03	0.03	0.03
Q30 (%)	92.63	92.63	92.51
GC content (%)	42.07	42.33	42.45
Gene number	46302	47166	46341
Transcripts number	75924	76516	74678

Then, data obtained from the three samples were subjected to pairwise comparisons, and DEGs were identified using the preset cutoffs. A total of 9216 DEGs (including 5604 solely expressed DEGs) were identified. When DT was compared to CT, 1227 genes were up-regulated, and 1962 genes were down-regulated. When RWT was compared to CT, 658 genes were found to be up-regulated and 1139 gene down-regulated. Different from the above two comparison groups, 2352 genes were up-regulated, and 1878 genes showed down-regulated mode when RWT was compared to DT. Down-regulated transcripts accounts for 62%, 63% and 44% of the total DEGs in above three comparison groups, respectively (S2 Table).

To screen the DEGs, two different analysis methods were applied in our study. First, we identified 20 up- and down-regulated genes whose expression differed significantly (fold change |log2| ≥1, corrected p-value <0.005) in DT *vs*. CT (Table 2), RWT *vs*. CT (Table 3) and RWT *vs*. DT (Table 4). Expression of heat shock protein (PGSC0003DMG400011197), aquaporins (PGSC0003DMG400026969, PGSC0003DMG400015275 and PGSC0003DMG400026463), bidirectional sugar transporters (PGSC0003DMG400032771 and PGSC0003DMG400008517) and lipid-transfer proteins (PGS C0003DMG400022295 and PGSC0003DMG400030587) differed significantly between DT and CT (Table 2). Whereas, expression of starch synthesis and accumulation, and water stimulus-responsive genes such as those encoding protein EARLY FLOWERING (PGSC0003DMG400001221), glucose-6-phosphate/phosphate translocator (PGSC0003DMG400005269), serine protease inhibitors (PGSC0003DMG400010128 and PGSC0003DMG400009512), granule-bound starch synthase (PGSC0003DMG400012111), ethylene-responsive transcription factor (PGSC0003DMG400012527), and gibberellin 3-beta-dioxygenase (PGSC0003DMG400005698) differed most significantly between RWT and CT (Table 3). Homologous gene of Cytochrome P450 (PGSC0003DMG400006368), Galactinol synthase (PGSC0003DMG400015490), bidirectional sugar transporter SWEET12 (PGSC0003DMG400032771), heat shock protein (PGSC0003DMG400011197), lipid-transfer proteins (PGSC0003DMG400030587), ASPG1 (PGSC0003DMG400037894) and BURP14 (PGSC0003DMG400027019) which related to stress signal transduction or carbohydrate synthesis and metabolism were significantly different expressed between RWT and DT groups (Table 4).

**Table 2:** List of genes showing significantly different expression in the comparison DT *vs*. CT.

Gene ID	Readcount DT	Readcount CT	Log_2_^Fold change^	q-value	Blast Swiss prot
PGSC0003DMG400012174	399.16	0	11.01	9.15E-55	-//-
PGSC0003DMG400011197	58.91	0	10.57	5.53E-09	Heat shock 70 kDa protein
PGSC0003DMG400026969	21.73	0	9.13	0.00023524	Probable aquaporin TIP3-2
PGSC0003DMG400026590	82.9	0	8.74	6.59E-16	Expansin-like B1
PGSC0003DMG400032771	40.67	0	8.04	1.29E-08	Bidirectional sugar transporter
PGSC0003DMG400015495	130.51	0	8.02	8.49E-27	-//-
PGSC0003DMG400008517	57.07	0	7.72	3.01E-12	Bidirectional sugar transporter
PGSC0003DMG400018766	28.23	0	7.51	2.09E-06	-//-
PGSC0003DMG400015275	11.96	0	6.68	0.0036329	Probable aquaporin TIP3-2
PGSC0003DMG400019320	46.12	0	6.63	1.12E-10	Premnaspirodiene oxygenase
PGSC0003DMG400002085	1.05	601.17	-9.17	7.90E-111	Pectinesterase/pectinesterase inhibitor 18
PGSC0003DMG400026575	0	80.14	-9.26	7.81E-15	Cationic peroxidase 1
PGSC0003DMG400022295	0	35.91	-9.68	1.52E-06	Putative lipid-transfer protein DIR1
PGSC0003DMG400005273	0	36.53	-9.71	1.26E-06	Peroxidase 3
PGSC0003DMG400002074	0	77.78	-9.8	2.12E-13	Pectinesterase/pectinesterase inhibitor 18
PGSC0003DMG400002108	0	163.19	-9.87	1.90E-27	-//-
PGSC0003DMG400010771	0	118.03	-10.4	1.94E-18	Probable pectate lyase 5
PGSC0003DMG400026463	0	356.71	-10.41	4.82E-55	Aquaporin TIP2-1
PGSC0003DMG400030587	0	126.28	-11.5	1.68E-16	Non-specific lipid-transfer protein 2

**Table 3:** List of genes showing significantly different expression in the comparison RWT *vs*. CT.

Gene ID	Readcount RWT	Readcount CT	Log_2_^Fold change^	q-value	Blast swiss prot
PGSC0003DMG400005269	1267.78	2.35	9.08	4.9453E^-227^	Glucose-6-phosphate/phosphate translocator 2, chloroplastic
PGSC0003DMG400025158	35.07	0	7.86	2.5423E^-07^	9-divinyl ether synthase
PGSC0003DMG400030925	22.28	0	6.20	0.000033552	-//-
PGSC0003DMG400009512	370.40	6.51	5.83	9.8857E^-83^	Serine protease inhibitor 1
PGSC0003DMG400022807	15.04	0	5.63	0.0012378	-//-
PGSC0003DMG400010128	578.44	12.41	5.54	6.6347E^-129^	Serine protease inhibitor 7
PGSC0003DMG400011323	41.67	1.02	5.35	2.2123E^-09^	Putative lipid-transfer protein DIR1
PGSC0003DMG400012111	1346.25	38.70	5.12	6.3024E^-296^	Granule-bound starch synthase 1, chloroplastic/amyloplastic
PGSC0003DMG400013516	41.31	1.48	4.81	5.0155E^-09^	Polygalacturonase At1g48100
PGSC0003DMG400028535	136.29	5.07	4.75	4.4972E^-29^	-//-
PGSC0003DMG400012494	0	59.92	-6.74	3.0461E^-14^	PGR5-like protein 1B, chloroplastic
PGSC0003DMG401026984	0	15.93	-7.05	0.00058233	BTB/POZ and TAZ domain-containing protein 1
PGSC0003DMG400012527	0	31.89	-7.32	1.9372E^-07^	Ethylene-responsive transcription factor ERF025
PGSC0003DMG400005698	0	16.61	-7.70	0.0006312	Gibberellin 3-beta-dioxygenase 3
PGSC0003DMG400007672	1.20	283.08	-7.88	3.4095E^-61^	-//-
PGSC0003DMG401028252	0	30.42	-8.57	2.5024E^-06^	Beta-fructofuranosidase, insoluble isoenzyme 1
Novel00218	0	34.77	-8.76	4.9726E^-07^	Phenylalanine N-monooxygenase
PGSC0003DMG400013481	0	40.59	-8.99	6.1882E^-08^	Probable inactive poly [ADP-ribose] polymerase SRO2
PGSC0003DMG400032247	0	67.34	-9.13	9.8393E^-13^	Vicilin-like antimicrobial peptides 2–1
PGSC0003DMG400001221	0	46.95	-10.20	9.4907E^-08^	Protein EARLY FLOWERING4

**Table 4:** List of genes showing significantly different expression in the comparison RWT *vs*. DT.

Gene ID	Readcount DT	Readcount RWT	Log_2_^Fold change^	q-value	Blast Swiss prot
PGSC0003DMG400012174	378.09	0	-12.22	2.2E^-43^	-//-
PGSC0003DMG400015495	123.62	0	-10.61	5.56E^-19^	-//-
PGSC0003DMG400014293	282.00	0	-10.48	1.21E^-43^	sp|Q04980|LTI65_ARATH Low-temperature-induced 65 kDa protein OS = Arabidopsis thaliana GN = LTI65 PE = 2 SV = 2//2.5331e-35
PGSC0003DMG400026590	78.53	0	-8.95	2.17E^-15^	sp|O23547|EXLB1_ARATH Expansin-like B1 OS = Arabidopsis thaliana GN = EXLB1 PE = 2 SV = 2//5.27796e-07
PGSC0003DMG400011197	55.80	0	-8.46	9.9E^-12^	sp|P26413|HSP70_SOYBN Heat shock 70 kDa protein OS = Glycine max GN = HSP70 PE = 3 SV = 1//4.92362e-50
PGSC0003DMG400032771	38.52	0	-7.60	5.85E^-09^	sp|O82587|SWT12_ARATH Bidirectional sugar transporter SWEET12 OS = Arabidopsis thaliana GN = SWEET12 PE = 2 SV = 1//1.31834e-26
Novel05792	60.63	0	-7.41	5.24E^-14^	sp|Q9M439|BCAT2_ARATH Branched-chain-amino-acid aminotransferase 2, chloroplastic OS = Arabidopsis thaliana GN = BCAT2 PE = 1 SV = 1//3.37023e-175
PGSC0003DMG400015490	22.81	0	-7.17	9.98E^-06^	sp|C7G304|GOLS2_SOLLC Galactinol synthase 2 OS = Solanum lycopersicum GN = GOLS2 PE = 2 SV = 1//1.32863e-42
PGSC0003DMG400028543	578.87	4.91	-6.88	4.7E^-135^	-//-
PGSC0003DMG400006368	12.36	0	-6.70	0.001778	sp|P24465|C71A1_PERAE Cytochrome P450 71A1 OS = Persea americana GN = CYP71A1 PE = 1 SV = 2//1.28232e-112
PGSC0003DMG400014850	0	245.89	9.95	1.16E^-39^	sp|P14009|14KD_DAUCA 14 kDa proline-rich protein DC2.15 OS = Daucus carota PE = 2 SV = 1//2.41216e-33
PGSC0003DMG400008980	0	85.25	10.01	6.01E^-14^	sp|P13917|7SB1_SOYBN Basic 7S globulin OS = Glycine max GN = BG PE = 1 SV = 2//4.91172e-60
PGSC0003DMG400014690	0	46.19	10.13	1.09E^-07^	-//-
PGSC0003DMG400027019	0	233.51	10.14	1.14E^-36^	sp|Q6K2M1|BURPE_ORYSJ BURP domain-containing protein 14 OS = Oryza sativa subsp. japonica GN = BURP14 PE = 2 SV = 2//1.71576e-37
PGSC0003DMG400022295	0	61.94	10.55	1.59E^-09^	sp|Q8W453|DIR1_ARATH Putative lipid-transfer protein DIR1 OS = Arabidopsis thaliana GN = DIR1 PE = 1 SV = 1//5.41356e-08
PGSC0003DMG400018917	0	153.88	10.86	8.22E^-22^	sp|Q08303|PPOA_SOLLC Polyphenol oxidase A, chloroplastic OS = Solanum lycopersicum PE = 3 SV = 2//0
PGSC0003DMG400037894	0	178.61	11.08	2.16E^-24^	sp|Q9LS40|ASPG1_ARATH Protein ASPARTIC PROTEASE IN GUARD CELL 1 OS = Arabidopsis thaliana GN = ASPG1 PE = 1 SV = 1//1.31192e-38
PGSC0003DMG400030587	0	111.53	11.40	1.24E^-14^	sp|P82353|NLTP2_PRUAR Non-specific lipid-transfer protein 2 OS = Prunus armeniaca PE = 1 SV = 1//1.21051e-14
PGSC0003DMG400008444	0	918.71	12.44	1.02E-^97^	-//-
PGSC0003DMG400005273	0	236.39	12.48	3.16E^-25^	sp|O23044|PER3_ARATH Peroxidase 3 OS = Arabidopsis thaliana GN = PER3 PE = 2 SV = 1//4.47243e-66

Second, of the identified DEGs, 263 of them exhibited opposite expression patterns between the two comparison treatments CT *vs*. DT and CT *vs*. RWT (S3 Table), that is, transcripts that were regulated in an opposite direction in DT and RWT compared to CT. The most significant 20 DEGs (with the greatest positive or negative changes) were genes homologous to those responsible for drought-stress response and regulation, such as *TAS14* (PGSC0003DMG400003530), *PP2C5* (PGSC0003DMG400027174), *ASPG1* (PGSC0003DMG400037894) and *BURP14* (PGSC0003DMG400027019) (Table 5). Additionally, prominent changes were also observed in the expression of some other stress response genes, such as those encoding WRKYs (PGSC0003DMG400011633, PGSC0003DMG400016769, PGSC0003DMG400028520 and PGSC0003DMG400021 895), ERFs (PGSC0003DMG400025797, PGSC0003DMG400008248 and PGSC0003D MG400008265), bHLHs (PGSC0003DMG400006394 and PGSC0003DMG400000599), cytochrome P450 (PGSC0003DMG401031520), auxin-responsive protein (PGSC0003DMG400006093), APRR2 (two-component response regulator-like, PGSC0003DMG400026587), GASA6 (gibberellin-regulated protein 6, PGSC0003DMG401019533), CML19 (putative calcium-binding protein, PGSC0003DMG400002993), SAMDC (S-adenosylmethionine decarboxylase proenzyme, PGSC0003DMG400010051), Abscisic acid 8'-hydroxylases (PGSC0003DMG402018475 and PGSC0003DMG400001 960), calcium-transporting ATPase (PGSC0003DMG400018942), F-box protein (PGSC0003DMG400026346) and ZOG1 (zeatin O-glucosyltransferases PGSC0003DMG400000432 and PGSC0003DMG400028331). In addition to the above genes, sixteen genes involved in starch accumulation and synthesis and tuber formation exhibited significantly different expression (S4 Table). Among them, six genes *PHYB* (PGSC0003DMG400027211), *LOX* (PGSC0003DMG400032155), *gibberellin 2-beta-dioxygenase* (PGSC0003DMG400026762), *gibberellin 20 oxidase* (PGSC0003DMG400001249) and aquaporin-encoding genes (PGSC0003DMG400007134 and PGSC0003DMG400011875) only showed differential expression between DT and CT, and two genes *LOX-*encoding gene (PGSC0003DMG400022894) and *GASA6* (PGSC0003DMG 401019533) exhibited opposite expression patterns.

**Table 5:** Twenty differentially expressed genes with most significant positive or negative changes.

Gene ID	Log2 Fold change (DT/CT)	Log2 Fold change (RWT/CT)	Range	Blast swiss prot
Novel05792	1.76	-5.68	7.44	Branched-chain-amino-acid aminotransferase 2, chloroplastic
PGSC0003DMG400028543	2.97	-3.94	6.91	-//-
PGSC0003DMG400002161	2.23	-3.39	5.62	-//-
PGSC0003DMG400000339	1.07	-4.54	5.60	Beta-galactosidase 1
PGSC0003DMG400016931	3.54	-1.88	5.42	-//-
PGSC0003DMG400003530	2.26	-3.04	5.30	Abscisic acid and environmental stress-inducible protein TAS14
PGSC0003DMG401033888	1.87	-3.25	5.12	Universal stress protein A-like protein
PGSC0003DMG400014487	2.19	-2.70	4.89	MOUSE RING finger and CHY zinc finger domain-containing protein 1
PGSC0003DMG400001518	2.12	-2.57	4.69	-//-
PGSC0003DMG400027174	3.27	-1.18	4.45	Probable protein phosphatase 2C 30
PGSC0003DMG400020481	-6.70	1.94	-8.63	14 kDa proline-rich protein DC2.15
PGSC0003DMG400026540	-7.90	1.04	-8.95	Uncharacterized protein At5g22580
PGSC0003DMG400006162	-5.95	3.03	-8.99	Fruit-specific protein
PGSC0003DMG400013680	-7.05	2.75	-9.80	Probable pectinesterase/pectinesterase inhibitor 12
PGSC0003DMG400014850	-6.91	3.01	-9.92	14 kDa proline-rich protein DC2.15
PGSC0003DMG400027019	-7.52	2.60	-10.11	BURP domain-containing protein 14
PGSC0003DMG400018917	-7.50	3.33	-10.83	Polyphenol oxidase A, chloroplastic
PGSC0003DMG400037894	-8.61	2.44	-11.05	Protein ASPARTIC PROTEASE IN GUARD CELL 1
PGSC0003DMG400008444	-9.09	3.32	-12.41	-//-
PGSC0003DMG400005273	-9.71	2.74	-12.45	Peroxidase 3

### qRT-PCR verification

**Fig 1:**
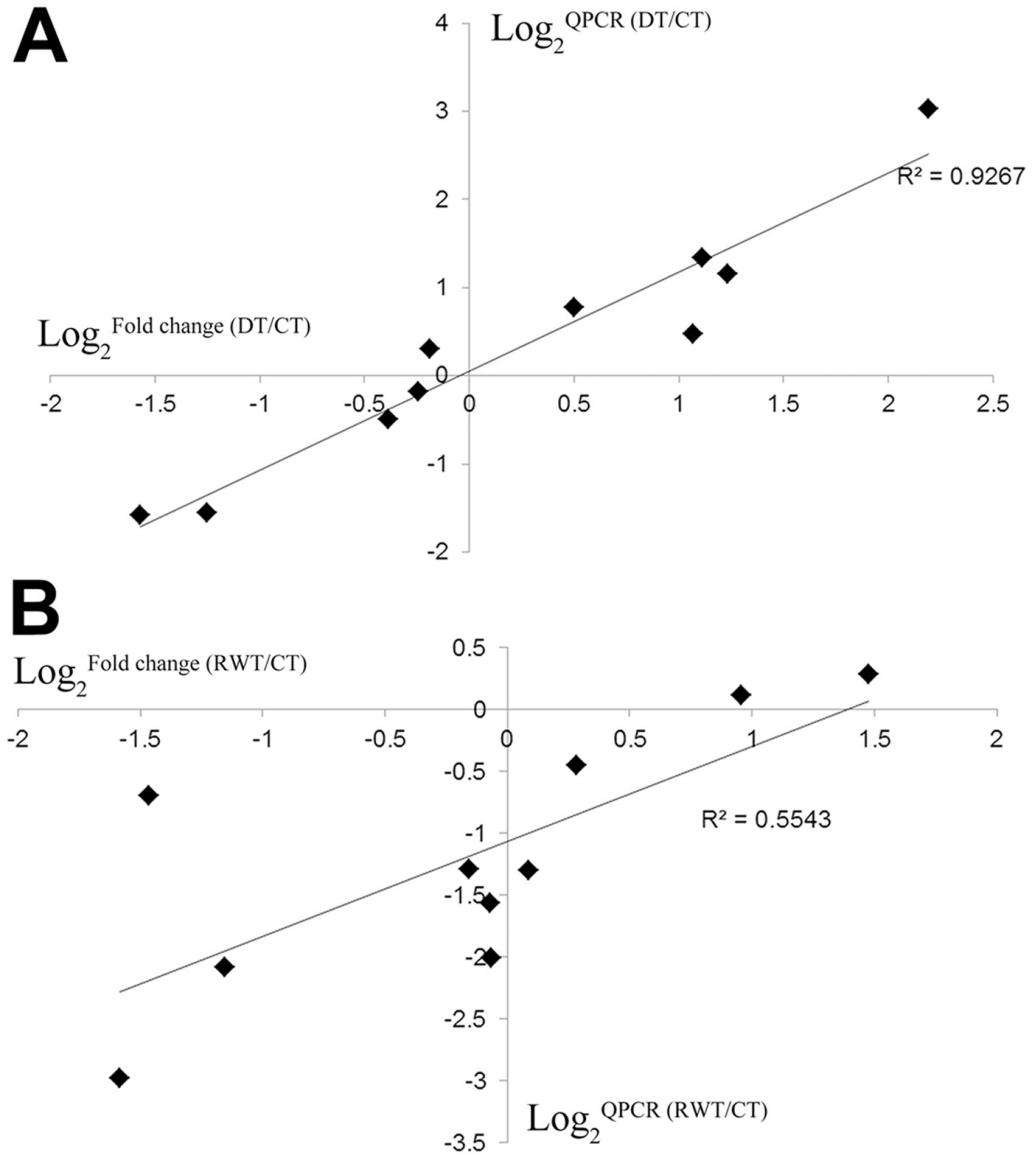
Verification of differentially expressed genes in DT *vs*. CT group (A) and in RW *vs*. CT group (B) by qRT-PCR.

To verify data validity, ten genes, including the target genes of interest, were selected from sequencing data, and qRT-PCR analyses were performed (S5 Table). In the comparison of DT and RWT, the correlation coefficients of gene expression trends in sequencing data and qRT-PCR results were 0.9267 and 0.5543, respectively (Fig 1). In particular, significant expression difference occurred in drought stress- and water stimulus-responsive genes before and after the treatment, including genes encoding *MYB60* (PGSC0003DMG400033043), *WRKY33* (PGSC0003DMG400011633) and *NAC072* (PGSC0003DMG400015342), thus supporting the validity of DEG data obtained.

### GO classification and KEGG pathway annotation analysis of DEGs

**Fig 2:**
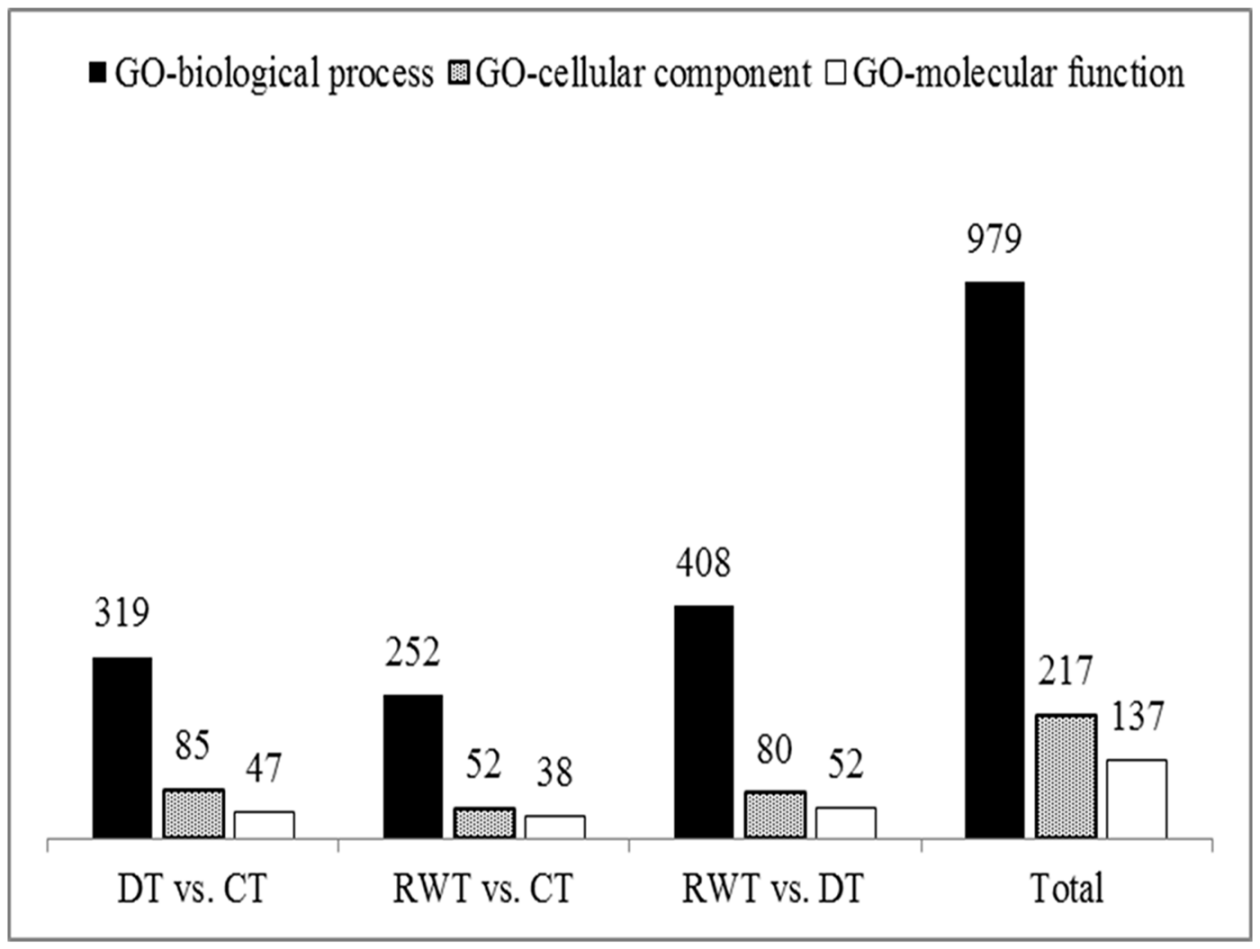
Gene Ontology (GO) categories of assembled differentially expressed genes in the comparison groups.

In order to investigate their functions and highlight metabolic pathways potentially related to drought tolerance, GO classification and KEGG pathway annotation analyses were performed on the identified DEGs. A total of 639 unique GO functional annotation terms were assigned to the identified DEGs (Fig 2). Of them, 979 were within the biological process category, accounting for 73.4% of the total GO functional annotation categories, and the rest were within the cellular component (217) and molecular function categories (137). Intra-group analysis (Table 6) revealed that in the comparison of CT and DT, the top 3 significantly enriched GO functional annotation categories were stress response (GO:0006950), macromolecular complex (GO:0032991) and structural constituent of ribosome (GO:0003735). These included 635 DEGs, of which 454 were down-regulated. In the comparison of CT and RWT, the top 3 significantly enriched GO functional annotation categories were translation (GO:0006412), ribosome (GO:000 5840) and structural constituent of ribosome (GO:0003735). These included 274 DEGs, of which, 256 were up-regulated genes. Clearly, DEGs were mainly down-regulated in DT and up-regulated in RWT. Similar to the RWT *vs*. CT group, the top 3 significantly enriched GO functional annotation categories in the comparison of DT and RWT were translation (GO:0006412), ribosome (GO:0005840) and structural constituent of ribosome (GO:0003735) either. However, each of the GO category include much more DEGs. Totally, this comparison group includes 713 DEGs, of which, 675 were up-regulated genes.

**Table 6:** Top 3 significantly enriched GO functional annotation categories.

	GO accession	Description	Term type	Corrected p-value	DEG item	Up	Down
DT *vs*. CT	GO:0006950	response to stress	biological process	6.85E^-16^	263	89	174
	GO:0032991	macromolecular complex	cellular component	1.65E^-20^	292	85	207
	GO:0003735	structural constituent of ribosome	molecular function	9.41E^-10^	80	7	73
				Total	635	181	454
RWT *vs*. CT	GO:0006412	translation	biological process	1.46E^-19^	104	91	13
	GO:0005840	ribosome	cellular component	9.04E^-24^	88	85	3
	GO:0003735	structural constituent of ribosome	molecular function	3.52E^-22^	82	80	2
				Total	274	256	18
RWT *vs*. DT	GO:0006412	translation	biological process	8.82E^-64^	255	235	20
	GO:0005840	ribosome	cellular component	2.76E^-97^	236	226	10
	GO:0003735	structural constituent of ribosome	molecular function	1.68E^-91^	222	214	8
				Total	713	675	38

**Fig 3:**
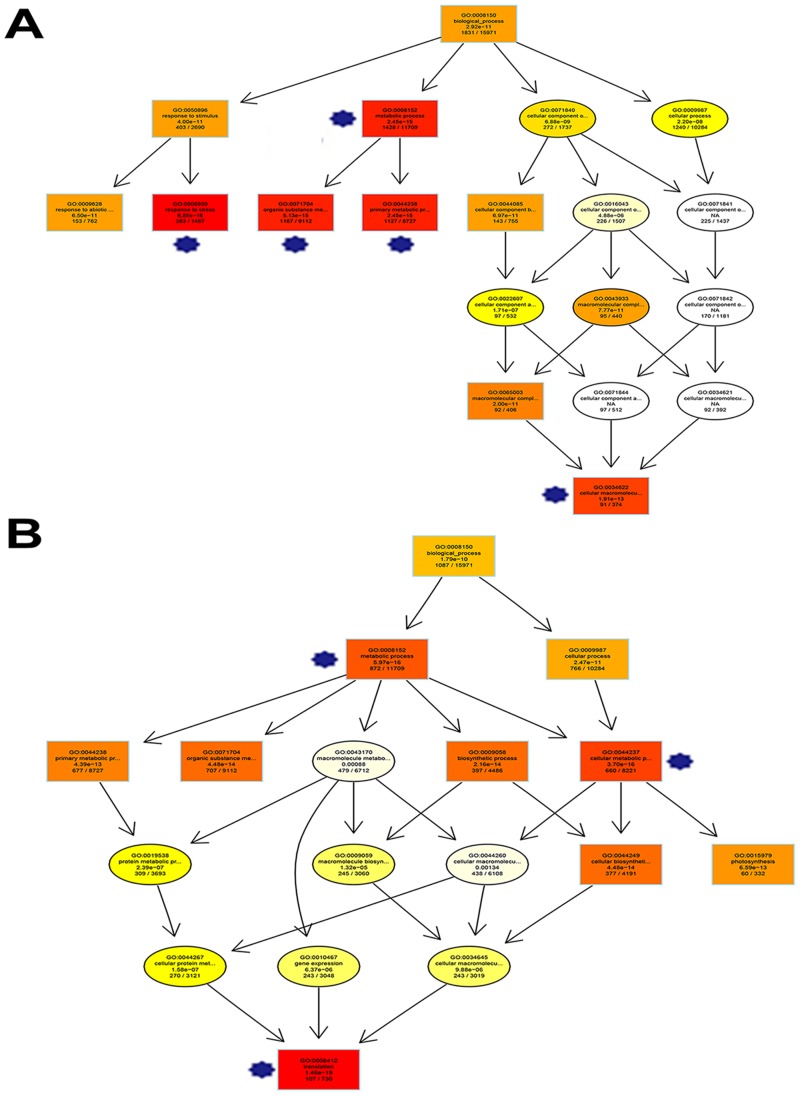
GO analysis of DT *vs*. CT group (A) and RWT *vs*. CT group (B) illustrated by Directed Acyclic Graph. A, *significantly enriched GO categories, including GO:0008152 (metabolic process, corrected p-value 2.45E^-15^), GO:0006950 (response to stress, corrected p-value 6.85E^-16^), GO:0071704 (organic substance metabolic process, corrected p-value 5.13E^-15^), GO:0044238 (primary metabolic process, corrected p-value 2.45E^-15^) and GO:0034622 (cellular macromolecular complex assembly, corrected p-value 1.91E^-13^). B, *significantly enriched GO categories, including GO: 0008152 (metabolic process, corrected p-value 5.97E^-16^), GO:0044237 (cellular metabolic process, corrected p-value 3.7E^-16^) and GO:0006412 (translation, corrected p-value 1.46E^-19^).

A directed acyclic graph (DAG) was used to illustrate the affiliation and functional scope of the 10 most significantly enriched GO categories. In the comparison of CT and DT (Fig 3A), DEGs were intensively enriched in the following five GO categories: metabolic process (GO:0008152), stress response (GO:0006950), organic substance metabolic process (GO:0071704), primary metabolic process (GO:0044238) and cellular macromolecular complex assembly (GO:0034622), which included 4076 DEGs. In the comparison of CT and RWT (Fig 3B), the top 3 significantly enriched GO categories were metabolic process (GO:0008152), cellular metabolic process (GO:0044237) and translation (GO:0006412).

To further identify the key regulatory genes related to drought- and water-stimulus responses, as well as genes involved in starch and sucrose metabolism, we focused on DEGs of the same GO terms but showed opposite expression regulation patterns between the two comparison treatments, that is, transcripts that were regulated in an opposite direction in DT and RWT compared to CT. A total number of 5 GO terms were related to starch and sucrose metabolism, and 3 GO terms were related to drought-stress and water-stimulus responses (S6 Table). Among these 8 GO terms, 60 DEGs showed opposite regulation patterns between DT and RWT. Further analysis revealed that 41 genes were down-regulated in DT and up-regulated in RWT, of which 37 were related to starch and sucrose metabolism, including homologous genes encoding *beta-galactosidase 1* (*BGAL1*), *UDP-glucose 4-epimerase* and *6-phosphogluconate dehydrogenase*. Additionally, 19 genes were up-regulated in DT and down-regulated in RWT, of which 13 were related to drought-stress and water-stimulus responses.

**Fig 4:**
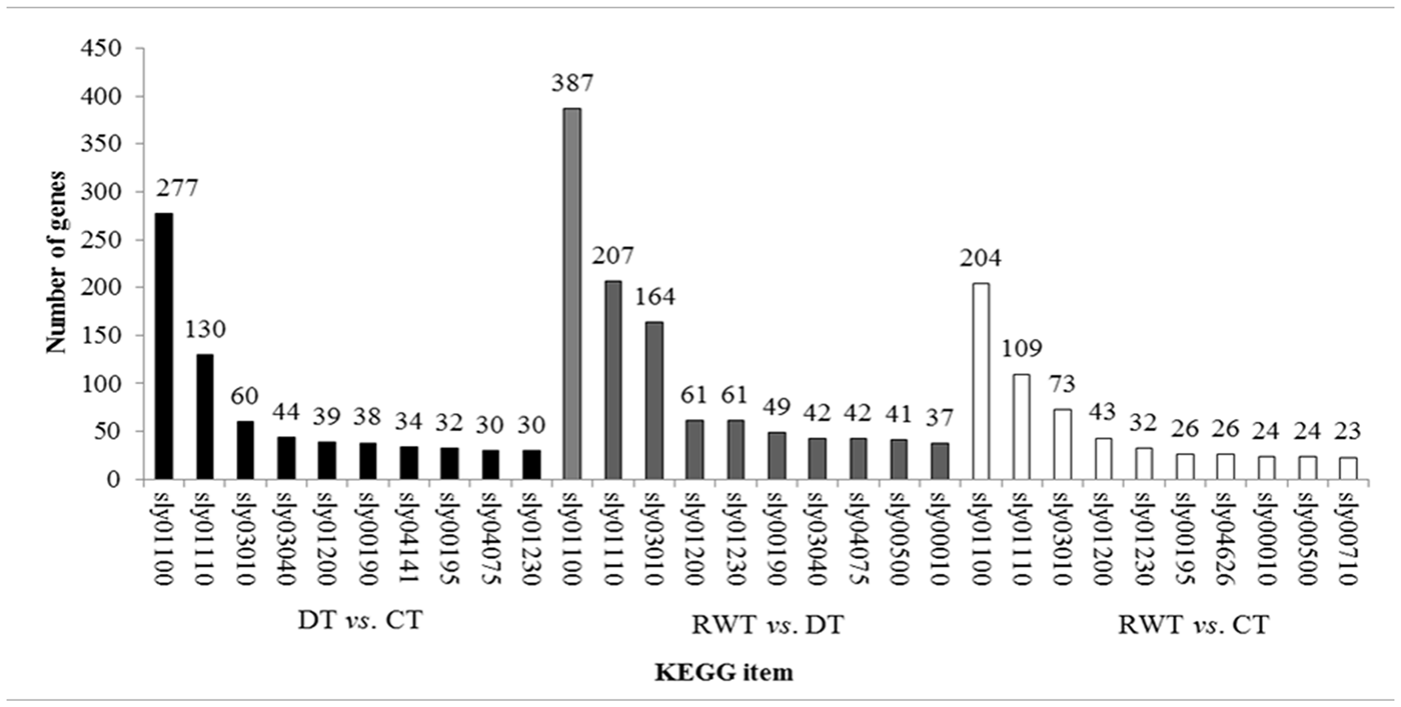
Top 10 statistically enriched KEGG pathways in the comparison groups. sly01100: Metabolic pathways, sly01110: Biosynthesis of secondary metabolites, sly03010: Ribosome, sly01200: Carbon metabolism, sly00190: Oxidative phosphorylation, sly04141: Protein processing in endoplasmic reticulum, sly00195: Photosynthesis, sly04075: Plant hormone signal transduction, sly01230: Biosynthesis of amino acids, sly00500: Starch and sucrose metabolism, sly04626: Plant-pathogen interaction, sly00010: Glycolysis/Gluconeogenesis, sly00710: Carbon fixation in photosynthetic organisms, sly03040: Spliceosome.

In the pathway annotation analysis, data was compared with the published KOBAS database. A total number of 4549 DEGs were enriched in 314 (114 unique) pathways. Intra-group analysis of each comparison was then performed to identify the 10 most significantly enriched pathways (Fig 4). Revealing DEGs were intensively enriched in 14 pathways. The metabolic pathways with the most significant DEGs enrichment were metabolic pathways (sly01100), biosynthesis of secondary metabolites (sly01110), ribosome (sly03010) and carbon cycle metabolism (sly01200), which included 868, 446, 297, and 143 DEGs, respectively.

Furthermore, we performed a detailed classification of KEGG pathways for the 263 genes which showed opposite expression regulation patterns between the two comparison treatments, revealing 66 DEGs annotated to KEGG pathways (S7 Table) including 16 metabolic pathways (Table 7). Of them, the most frequently associated pathways were metabolic pathways (21), followed by ribosome (18). Only 3 enriched DEGs were associated with carotenoid biosynthesis and plant hormone signal transduction pathways (S1A and S1B Fig). These included PGSC0003DMG402018475 (encoding *abscisic acid 8'-hydroxylase 1-like*, *CYP707A2*), *PGSC0003DMG400001960* (encoding *abscisic acid 8'-hydroxylase 1-like*) and PGSC0003DMG400028897 (encoding *beta-carotene hydroxylase*) involved in carotenoid biosynthesis pathway, and PGSC0003DMG400008011 (encoding *AREB-like protein*, AREB), PGSC0003DMG40 1025624 (encoding *two-component response regulator ARR2-like*) and PGSC0003DM G400006093 (encoding *auxin-responsive protein*, AUX) associated with plant hormone signal transduction pathway. The above results suggest that DEGs with opposite expression regulation patterns were mainly involved in drought- and water-stimulus response and regulation via the carotenoid biosynthesis pathway, which affect the synthesis and signal transduction pathways of other endogenous hormones (e.g., abscisic acid and indole acetic acid).

**Table 7:** The number of differentially expressed genes with opposite expression patterns annotated to KEGG pathways.

KEGG pathway item	DEGs number
Metabolic pathways	21
Ribosome	18
Plant-pathogen interaction	4
Carotenoid biosynthesis	3
Plant hormone signal transduction	3
Stilbenoid, diarylheptanoid and gingerol biosynthesis	2
Ubiquitin mediated proteolysis	2
Sulfur metabolism	2
Galactose metabolism	2
Steroid biosynthesis	2
mRNA surveillance pathway	2
Phagosome	1
Vitamin B6 metabolism	1
Biosynthesis of secondary metabolites	1
Photosynthesis	1
Ribosome biogenesis in eukaryotes	1
Total	66

### Drought stress and water stimulus responsive TFs

TF-encoding genes are key factors in the process of stress signal perception and transduction. Based on the sequencing analyses, a total number of 1630, 1527 and 1596 TF-encoding genes were detected in CT, DT and RWT, respectively (Table 8). In the comparison to the control treatment, both drought and re-watering treatments reduced the number of TF-encoding genes detected. In the comparison between DT and RWT with CT, a total number of 1019 and 866 TF-encoding DEGs were identified, respectively.

**Table 8:** Transcription factors (TFs) in sequencing libraries.

	Normal control (CT)	Drought stress (DT)	Re-watering after drought stress (RWT)
TFs	1630	1527	1596
	DT *vs*. CT	RWT *vs*. CT	RWT *vs*. DT
Total differentially expressed TFs	1019	866	1018
Up-regulated differentially expressed TFs	336	442	648
Down-regulated differentially expressed TFs	683	424	370

**Fig 5:**
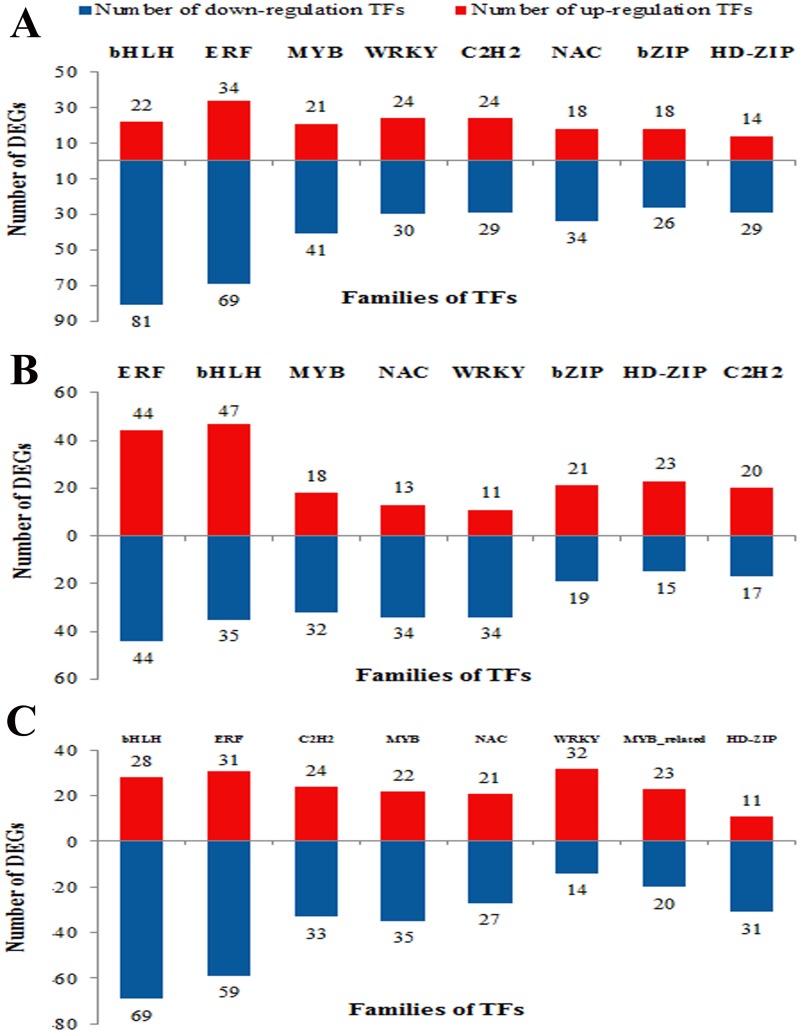
Top 10 families of differentially expressed transcription factors in DT *vs*. CT group (A), in RWT *vs*. CT group (B) and RWT *vs*. DT (C).

Further analyses revealed that in the comparison between DT and CT, the identified 1019 DEGs fell into 50 TF families, and most of DEGs (268) belong to bHLH, ERF and MYB families (S8 Table). Similarly, in the comparison between RWT and CT, the identified DEGs were involved in a total of 52 TF families, and most of them (230) also belong to ERF, bHLH, and MYB families. When it comes to RWT *vs*. DT, 1018 DEGs were involved in a total of 51 TF families. bHLH (97), ERF (90), C2H2 (57) and MYB (57) were the top 4 families with the most differently expressed genes. Overall (S8 Table, Fig 5A, 5B and 5C), significant changes occurred in the transcripts of the following eight TF families: bHLH (282 DEGs), ERF (281 DEGs), MYB (169 DEGs), C2H2 (147), NAC (147 DEGs), WRKY (145 DEGs), HD-ZIP (123) and bZIP (84 DEGs). DEGs belonging to the above eight transcription factor families accounted for almost 50% of the total differentially expressed TFs detected in the DT and RWT treatments, respectively.

We also analyzed the quantity relationship of up- and down-regulated TFs in 3 comparison groups (S8 Table). In the comparison between DT and CT, the number of down-regulated DEGs was approximately 2-fold that of the up-regulated ones among the 1019 differentially expressed TFs. The largest number of down-regulated DEGs was found in the bHLH family, while that of up-regulated DEGs was mostly found in the ERF family. Similar with comparison between DT and CT, the number of down-regulation DEGs in RWT *vs*. DT exhibited almost twice as much as the number of up-regulation TFs. The bHLH family contained 69 decreased expression of TFs and the ERF family included 31 increased expression of DEGs. In the comparison between RWT and CT, the number of up- or down-regulated DEGs was about the same among the 866 differentially expressed TFs. The largest number of down-regulated DEGs was found in the ERF family, whereas the largest number of up-regulated ones was found in the bHLH family.

## Discussion

Rapid growth of global population and decrease of arable land have presented a serious challenge for food production. Potato is a non grain crop and one of the most important sources for food and industry uses worldwide. One of the most essential approaches is to breed new plant species which can grow on uncultivated lands. Understanding how plants including potato respond to water-deficit stress will facilitate the breeding of drought tolerant plants. In the present study, we employed high-throughput sequencing technology to profile the transcriptome changes in the stolon tissues of potato undergoing water-deficit stress and re-watering treatment. A total number of 3189, 1797 and 4230 DEGs (S2 Table), including 1630, 1527 and 1596 transcriptional factor-encoding DEGs (Table 8), were identified in the comparisons within CT, DT and RWT, respectively. These DEGs were classified into 639 unique gene ontology functional annotation terms and were involved in 114 Kyoto Encyclopedia of Genes and Genomes pathways.

In terms of the current reports, cDNA microarrays and target metabolite analysis were performed on leaves of two different drought-tolerance potato clones (*Solanum tuberosum* subsp. *andigenum*) [2]. Gene expression analysis revealed that photosynthesis, photorespiration and carbohydrate-related gene, coupled with SPS and SS, showed more drastically down regulated in drought-tolerance clone than the expression in drought-sensitive clone. Zhang *et al*. [18] reported transcriptome dynamics of potato leaves under drought stress with high throughput sequencing platform and according to their report the DEGs were mainly annotated as genes coding for metabolism alteration, osmolite adjustment, cell rescue and gene regulation. Peripheral-type benzodiazepine receptor and polygalacturonase noncatalytic subunit AroGP3 were the one of the ten most up-regulated and the ten most down-regulated genes, respectively. Unlike previous studies, we took stolon tissues, one of the major agronomic traits, as sequencing material to examine transcriptome changes under two treatments and identified more significantly altered DEGs associated with drought stress (Tables 2, 3 and 4), starch synthesis and transfer (S4 Table), and water stimulus response (S6 Table). The difference of DEGs identified in these studies could be due to the different material properties, treatments, sampling strategies and genetic backgrounds of plants.

Many functional and regulatory genes are responsive to water deficit stress [19, 20]. The main role of these functional genes is to maintain intracellular water and ion homeostasis, membrane structural stability, and reconstruction of primary and secondary metabolism [21], whereas regulatory genes (e.g., calcium ions, receptor protein kinases, and TFs) are involved in stress response by regulating signal transduction and metabolic pathways [22, 23]. Heat shock responsive genes was one of the functional gene that facilitate protein refolding and stabilize polypeptides and membranes under stress condition by preventing protein from aggregation, protecting non-native enzymes from degradation and assisting protein refolding [24, 25]. Expression induction of these genes under drought stress has been reported in barley [26] and rice [27]. Our data showed different expression of heat shock protein-encoding genes (Tables 2 and 3), indicating that they were also directly involved in regulating drought-stress responses in potato.

Aquaporins, which facilitate transmembrane transport of small molecules such as water, have been showed to be involved in response to drought and salt stress, and to ABA treatment [28, 29]. In our data, aquaporin genes (Tables 2 and 3, S4 Table) were significantly expressed in Ningshu 4 stolons under drought stress condition. Similar expression pattern was also observed in Arabidopsis [28], chickpea (*Cicer arietinum* L.) [30], foxtail millet (*Setaria italica* L.) [31] and maize [32]. Additionally, ScPIP2a, an aquaporin, was reported to act in photoperiod perception, generating flower-inducing signals in the leaves [33]. The existing researches indicated these proteins could play an important role in water stimulus response and the maintenance of intracellular osmotic potential stability and organelle integrity. Therefore, we hypothesize that aquaporin(s) may play a similar regulatory role in potato tuber bulking and growth under drought stress.

Potato tuber formation is a complex biological process governed by both environmental factors and genes [34]. Cell division and expansion, together with the associated starch and protein accumulation, are the major events of tuber bulking in potato [35]. Photoperiod perception in leaves initiates tuberization in the subapical region of the underground stolons [36]. Dioxygenases, which catalyze the oxygenation of polyunsaturated fatty acids (LOXs) such as linoleic and linolenic acids, contribute to 9(S)-hydroperoxy linolenic acid (9(S)-HPOT) and 13(S)-hydroperoxy linolenic acid (13(S)-HPOT) accumulation [37]. Jasmonic acid is synthesized from 13(S)-HPOT and then metabolized to tuberonic acid and tuberonic acid glucoside, both of which induce tuber formation [38]. In this study, expression of *PHYB*, GA and *LOX-*encoding gene (S4 Table) was significantly different in drought and re-watering plants, suggesting that these genes could function in regulating starch accumulation and tuber formation of potato plants.

As an important plant hormone, ABA plays important roles in plant response to drought stress by inducing the expression of TF-, heat shock protein-, transporter-, and osmotic regulator-encoding genes downstream of stress signaling pathways in both ABA-dependent and ABA-independent manners [19]. We observed that expression of ABA anabolism- or signal transduction-related genes such as *abscisic acid 8'-hydroxylase 1-like*, *Beta-carotene hydroxylase*, *AREB*, and *PP2C5* was all significantly altered during drought stress and re-watering treatment (Table 5, S3 Table), indicating that ABA and anabolism related genes were also important regulators for drought-stress and re-watering stimulus-response in potato. This hypothesis is also supported by previous findings in corn (*Zea mays*) and cotton (*Gossypium hirsutum*) [24, 32]. In addition, expression of some gene involved in auxin, cytokinin and gibberellin anabolism was significantly affected (S3 Table, S1 Fig), which, together with ABA, jointly regulates the response of potato stolons to drought stress and re-watering. Similar results were also found in Chinese cabbage (*Brassica rapa* ssp. pekinensis) [39] and barley (*Hordeum vulgare*) [40]. Collectively, our findings demonstrate the existence of a synergistic and antagonistic cross talk at endogenous hormone level in potato during the response to drought stress and re-watering treatment.

Many TFs, such as ERF [41], MYB [42], WRKY [43] and bHLH, act as key regulators in signal transduction pathways involved in plant response to drought stress [19, 20]. Consistently, we observed four TF-encoding genes, WRKY, bHLH, ERF and MYB, were differentially expressed under drought stress and re-watering conditions (Tables 2 and 3, S3 Table). In particular, genes encoding WRKY, bHLH, and ERF showed opposite expression alterations under either condition, indicating that TF-encoding genes may have important functions in regulating the response to drought stress and re-watering stimulus in potato. Meanwhile, expression of genes mediated by them, such as those encoding transporter, osmotic adaptor, and those involved in fatty acid metabolism pathway (Tables 2 and 3, S3 Table), was also up- or down-regulated. Therefore, we postulate that drought stress response in potato stolons is similar to that in cotton as proposed by Padmalatha *et al* [24]. That is, potato stolons mediate the synthesis and signal transduction of ABA and the other endogenous hormones, and thereby influence the expression of downstream genes related to heat shock proteins, fatty acid metabolism and starch anabolism. Consequently, this mechanism further realizes drought- and water-stimulus response and regulation.

Based on DEGs detection, enriched biological processes and metabolism pathways analysis, our results provide a probable insight into the molecular mechanism in potato tuber response to drought stress and water-stimulus conditions. Our data shown in this work will provide more direct evidence and information for future study on the interaction between key genes involved in various metabolic pathways under drought stress condition. More detailed studies on the components involved in these pathways will help to understand the precise regulation process and mechanism related to drought stress response in potato, as well as for the molecular breeding of drought tolerant plants.

## Supporting Information

S1 Fig Enriched differentially expressed genes in the metabolic pathway of carotenoid biosynthesis (A) and plant hormone signal transduction (B) in the comparison groups. (TIF)

S1 Table Primers used for qRT-PCR. (XLS)

S2 Table The number and regulation pattern of differentially expressed genes. (XLS)

S3 Table Genes with opposite expression patterns between the comparisons DT *vs*. CT and RWT *vs*. CT. (XLS)

S4 Table Differentially expressed genes related to starch synthesis and accumulation and tuber formation. (XLS)

S5 Table qRT-PCR verification data. (XLS)

S6 Table Genes of the same GO annotation items with opposite differential expression patterns between comparison groups DT *vs*. CT and RWT *vs*. CT. (XLS)

S7 Table Significantly differentially expressed genes annotated to KEGG pathways. (XLS)

S8 Table Transcription factor (TF) families related to differentially expressed genes in comparison groups. (XLS)

## References

[1] Vasquez-Robinet C, Mane SP, Ulanov AV, Watkinson JI, Stromberg VK, De Koeyer D, et al (2008) Physiological and molecular adaptations to drought in Andean potato genotypes. J Exp Bot 59(8): 2109–2123. 10.1093/jxb/ern073 18535297 PMC2413284

[2] Evers D, Lefèvre I, Legay S, Lamoureux D, Hausman J-F, Rosales ROG, et al (2010) Identification of drought-responsive compounds in potato through a combined transcriptomic and targeted metabolite approach. J Exp Bot 61(9): 2327–2343. 10.1093/jxb/erq060 20406784

[3] Bhargava S, Sawant K. (2013) Drought stress adaptation: metabolic adjustment and regulation of gene expression. Plant Breeding 132(1): 21–32. 10.1111/pbr.12004 .24843434

[4] Anithakumari AM, Nataraja K, Visser RF, Linden CG. (2012) Genetic dissection of drought tolerance and recovery potential by quantitative trait locus mapping of a diploid potato population. Mol Breed 30(3): 1413–1429. 10.1007/s11032-012-9728-5 23024597 PMC3460171

[5] Xu X, Pan S, Cheng S, Zhang B, Mu D, Ni P, et al (2011) Genome sequence and analysis of the tuber crop potato. Nature 475(7355): 189–195. 10.1038/nature10158 21743474

[6] Massa AN, Childs KL, Lin H, Bryan GJ, Giuliano G, Buell CR. (2011) The Transcriptome of the Reference Potato Genome *Solanum tuberosum* Group Phureja Clone DM1-3 516R44. PLoS ONE 6(10): e26801 10.1371/journal.pone.0026801 22046362 PMC3203163

[7] Livaja M, Wang Y, Wieckhorst S, Haseneyer G, Seidel M, Hahn V, et al (2013) BSTA: a targeted approach combines bulked segregant analysis with next-generation sequencing and *de novo* transcriptome assembly for SNP discovery in sunflower. BMC Genomics 14(1): 628–637. 10.1186/1471-2164-14-628 24330545 PMC3848877

[8] Liu G-T, Wang J-F, Cramer G, Dai Z-W, Duan W, Xu H-G, et al (2012) Transcriptomic analysis of grape (*Vitis vinifera* L.) leaves during and after recovery from heat stress. BMC Plant Biol 12(1): 174–183. 10.1186/1471-2229-12-174 23016701 PMC3497578

[9] Lu X, Chen D, Shu D, Zhang Z, Wang W, Klukas C, et al (2013) The Differential Transcription Network between Embryo and Endosperm in the Early Developing Maize Seed. Plant Physiol 162(1): 440–455. 10.1104/pp.113.214874 23478895 PMC3641222

[10] Mizuno H, Kawahigashi H, Kawahara Y, Kanamori H, Ogata J, Minami H, et al (2012) Global transcriptome analysis reveals distinct expression among duplicated genes during sorghum-*Bipolaris sorghicola* interaction. BMC Plant Biol 12(1): 121–135. 10.1186/1471-2229-12-121 . 22838966 PMC3480847

[11] Trapnell C, Pachter L, Salzberg SL. (2009) TopHat: discovering splice junctions with RNA-Seq. Bioinformatics 25(9): 1105–1111. 10.1093/bioinformatics/btp120 19289445 PMC2672628

[12] Mortazavi A, Williams BA, McCue K, Schaeffer L, Wold B. (2008) Mapping and quantifying mammalian transcriptomes by RNA-Seq. Nat Methods 5(7): 621–628. 10.1038/nmeth.1226 18516045 PMC13303166

[13] Wang L, Feng Z, Wang X, Wang X, Zhang X. (2010) DEGseq: an R package for identifying differentially expressed genes from RNA-seq data. Bioinformatics 26(1): 136–138. 10.1093/bioinformatics/btp612 19855105

[14] Young MD, Wakefield MJ, Smyth GK, Oshlack A. (2010) Gene ontology analysis for RNA-seq: accounting for selection bias. Genome Biology 11(2): R14–R14. 10.1186/gb-2010-11-2-r14 20132535 PMC2872874

[15] Kanehisa M, Araki M, Goto S, Hattori M, Hirakawa M, Itoh M, et al (2008) KEGG for linking genomes to life and the environment. Nucleic Acids Res 36(suppl 1): D480–D484. 10.1093/nar/gkm882 18077471 PMC2238879

[16] Nicot N, Hausman J-F, Hoffmann L, Evers D. (2005) Housekeeping gene selection for real-time RT-PCR normalization in potato during biotic and abiotic stress. J Exp Bot 56(421): 2907–2914. 10.1093/jxb/eri285 16188960

[17] Livak KJ, Schmittgen TD. (2001) Analysis of Relative Gene Expression Data Using Real-Time Quantitative PCR and the 2^-ΔΔCT^ Method. Methods 25(4): 402–408. 10.1006/meth.2001.1262 11846609

[18] Zhang N, Liu B, Ma C, Zhang G, Chang J, Si H, et al (2014) Transcriptome characterization and sequencing-based identification of drought-responsive genes in potato. Mol Biol Rep 41(1): 505–517. 10.1007/s11033-013-2886-7 24293150

[19] Shinozaki K, Yamaguchi-Shinozaki K. (2007) Gene networks involved in drought stress response and tolerance. J Exp Bot 58(2): 221–227. 10.1093/jxb/erl164 17075077

[20] Golldack D, Lüking I, Yang O. (2011) Plant tolerance to drought and salinity: stress regulating transcription factors and their functional significance in the cellular transcriptional network. Plant Cell Rep 30(8): 1383–1391. 10.1007/s00299-011-1068-0 . 21476089

[21] Villar E, Klopp C, Noirot C, Novaes E, Kirst M, Plomion C, et al (2011) RNA-Seq reveals genotype-specific molecular responses to water deficit in eucalyptus. BMC Genomics 12(1): 538–555. 10.1186/1471-2164-12-538 22047139 PMC3248028

[22] Marshall A, Aalen RB, Audenaert D, Beeckman T, Broadley MR, Butenko MA, et al (2012) Tackling Drought Stress: RECEPTOR-LIKE KINASES Present New Approaches. Plant Cell 24(6): 2262–2278. 10.1105/tpc.112.096677 22693282 PMC3406892

[23] Hussain SS, Kayani MA, Amjad M. (2011) Transcription factors as tools to engineer enhanced drought stress tolerance in plants. Biotechnol Prog 27(2): 297–306. 10.1002/btpr.514 21302367

[24] Padmalatha K, Dhandapani G, Kanakachari M, Kumar S, Dass A, Patil D, et al (2012) Genome-wide transcriptomic analysis of cotton under drought stress reveal significant down-regulation of genes and pathways involved in fibre elongation and up-regulation of defense responsive genes. Plant Mol Biol 78(3): 223–246. 10.1007/s11103-011-9857-y 22143977

[25] Busch W, Wunderlich M, Schöffl F. (2005) Identification of novel heat shock factor-dependent genes and biochemical pathways in *Arabidopsis thaliana* . Plant J 41(1): 1–14. 10.1111/j.1365-313X.2004.02272.x 15610345

[26] Talamè V, Ozturk NZ, Bohnert HJ, Tuberosa R. (2007) Barley transcript profiles under dehydration shock and drought stress treatments: a comparative analysis. J Exp Bot 58(2): 229–240. 10.1093/jxb/erl163 17110587

[27] Rabbani MA, Maruyama K, Abe H, Khan MA, Katsura K, Ito Y, et al (2003) Monitoring Expression Profiles of Rice Genes under Cold, Drought, and High-Salinity Stresses and Abscisic Acid Application Using cDNA Microarray and RNA Gel-Blot Analyses. Plant Physiol 133(4): 1755–1767. 10.1104/pp.103.025742 14645724 PMC300730

[28] Alexandersson E, Danielson JÅH, Råde J, Moparthi VK, Fontes M, Kjellbom P, et al (2010) Transcriptional regulation of aquaporins in accessions of *Arabidopsis* in response to drought stress. Plant J 61(4): 650–660. 10.1111/j.1365-313X.2009.04087.x 19947979

[29] Alexandersson E, Fraysse L, Sjövall-Larsen S, Gustavsson S, Fellert M, Karlsson M, et al (2005) Whole Gene Family Expression and Drought Stress Regulation of Aquaporins. Plant Mol Biol 59(3): 469–484. 10.1007/s11103-005-0352-1 16235111

[30] Jain D, Chattopadhyay D. (2010) Analysis of gene expression in response to water deficit of chickpea (*Cicer arietinum* L.) varieties differing in drought tolerance. BMC Plant Biol 10(1): 24 10.1186/1471-2229-10-24 20144227 PMC2831037

[31] Lata C, Sahu PP, Prasad M. (2010) Comparative transcriptome analysis of differentially expressed genes in foxtail millet (*Setaria italica* L.) during dehydration stress. Biochem Biophys Res Commun 393(4): 720–727. 10.1016/j.bbrc.2010.02.068 20171162

[32] Hayano-Kanashiro C, Calderón-Vázquez C, Ibarra-Laclette E, Herrera-Estrella L, Simpson J. (2009) Analysis of Gene Expression and Physiological Responses in Three Mexican Maize Landraces under Drought Stress and Recovery Irrigation. PLoS ONE 4(10): e7531 10.1371/journal.pone.0007531 19888455 PMC2766256

[33] O'Brien M, Bertrand C, Matton D. (2002) Characterization of a fertilization-induced and developmentally regulated plasma-membrane aquaporin expressed in reproductive tissues, in the wild potato *Solanum chacoense* Bitt. Planta 215(3): 485–493. 10.1007/s00425-002-0770-0 12111232

[34] Kloosterman B, De Koeyer D, Griffiths R, Flinn B, Steuernagel B, Scholz U, et al (2008) Genes driving potato tuber initiation and growth: identification based on transcriptional changes using the POCI array. Funct Integr Genomics 8(4): 329–340. 10.1007/s10142-008-0083-x 18504629

[35] Sarkar D. (2008) The signal transduction pathways controlling in planta tuberization in potato: an emerging synthesis. Plant Cell Rep 27(1): 1–8. 10.1007/s00299-007-0457-x 17906863

[36] Kloosterman B, Vorst O, Hall RD, Visser RGF, Bachem CW. (2005) Tuber on a chip: differential gene expression during potato tuber development. Plant Biotechnol J 3(5): 505–519. 10.1111/j.1467-7652.2005.00141.x 17173637

[37] Nam K-H, Kong F, Matsuura H, Takahashi K, Nabeta K, Yoshihara T. (2008) Temperature regulates tuber-inducing lipoxygenase-derived metabolites in potato (*Solanum tuberosum*). J Plant Physiol 165(2): 233–238. 10.1016/j.jplph.2007.04.003 17643553

[38] Kolomiets MV, Hannapel DJ, Chen H, Tymeson M, Gladon RJ. (2001) Lipoxygenase Is Involved in the Control of Potato Tuber Development. Plant Cell 13(3): 613–626. 10.1105/tpc.13.3.613 11251100 PMC135504

[39] Liu Z, Lv Y, Zhang M, Liu Y, Kong L, Zou M, et al (2013) Identification, expression, and comparative genomic analysis of the IPT and CKX gene families in Chinese cabbage (*Brassica rapa* ssp. *pekinensis*). BMC Genomics 14(1): 594–613. 10.1186/1471-2164-14-594 24001366 PMC3766048

[40] Seiler C, Harshavardhan VT, Reddy PS, Hensel G, Kumlehn J, Eschen-Lippold L, et al (2014) Abscisic Acid Flux Alterations Result in Differential Abscisic Acid Signaling Responses and Impact Assimilation Efficiency in Barley under Terminal Drought Stress. Plant Physiol 164(4): 1677–1696. 10.1104/pp.113.229062 24610749 PMC3982733

[41] Zhang G, Chen M, Li L, Xu Z, Chen X, Guo J, et al (2009) Overexpression of the soybean *GmERF3* gene, an AP2/ERF type transcription factor for increased tolerances to salt, drought, and diseases in transgenic tobacco. J Exp Bot 60(13): 3781–3796. 10.1093/jxb/erp214 19602544 PMC2736888

[42] Shin D, Moon S-J, Han S, Kim B-G, Park SR, Lee S-K, et al (2011) Expression of *StMYB1R-1*, a Novel Potato Single MYB-Like Domain Transcription Factor, Increases Drought Tolerance. Plant Physiol 155(1): 421–432. 10.1104/pp.110.163634 21030505 PMC3075785

[43] Ren X, Chen Z, Liu Y, Zhang H, Zhang M, Liu Q, et al (2010) ABO3, a WRKY transcription factor, mediates plant responses to abscisic acid and drought tolerance in Arabidopsis. Plant J 63(3): 417–429. 10.1111/j.1365-313X.2010.04248.x . 20487379 PMC3117930

