# Supplementary figures and images for: Transcriptome Profiling of the Potato (Solanum tuberosum L.) Plant under Drought Stress and Water-Stimulus Conditions
Source: PLoS One. 2015 May 26;10(5):e0128041. doi: 10.1371/journal.pone.0128041 (PMC4444143; doi:10.1371/journal.pone.0128041)

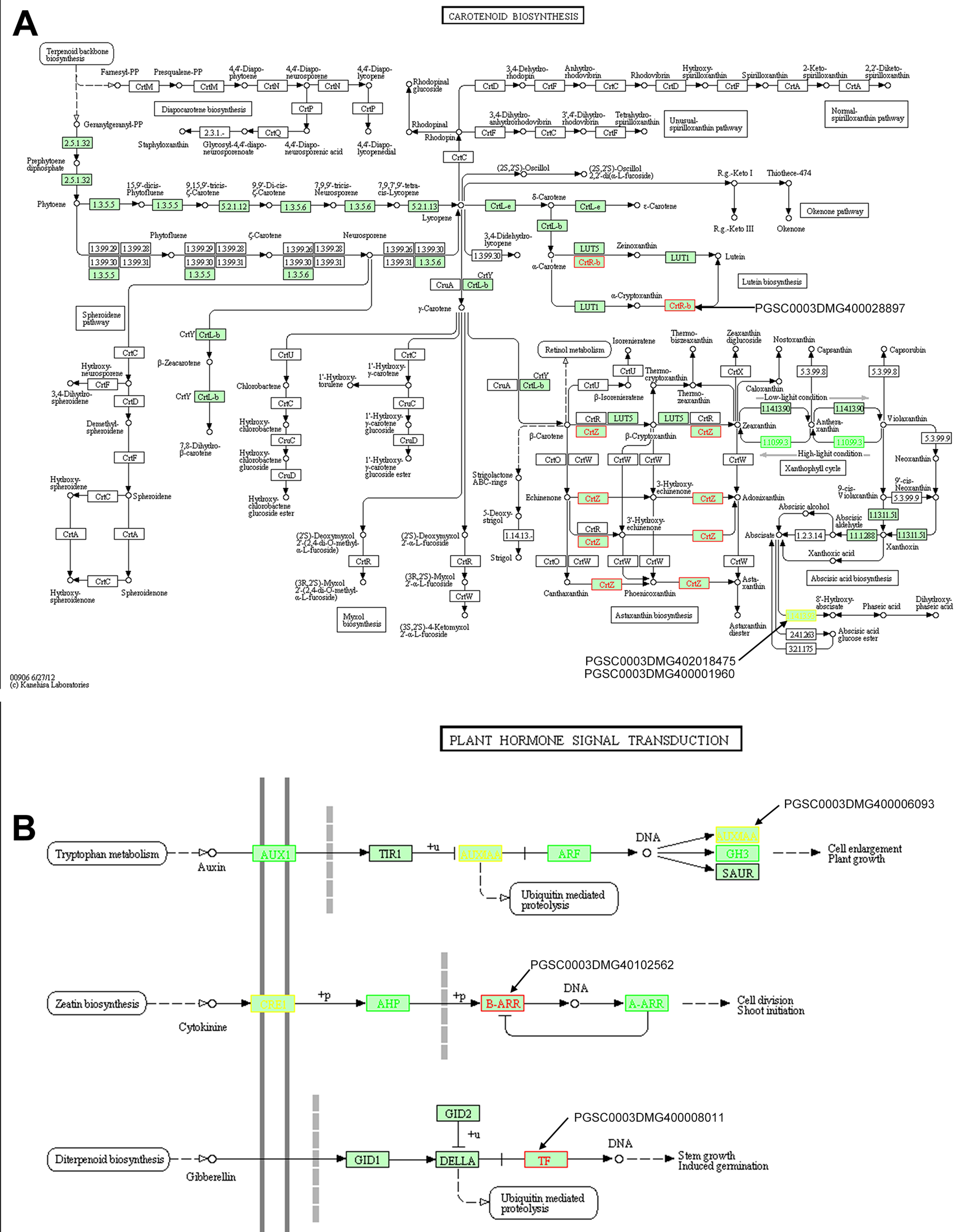

Supplement: S1 Fig — (TIF) [file pone.0128041.s001.tif]
